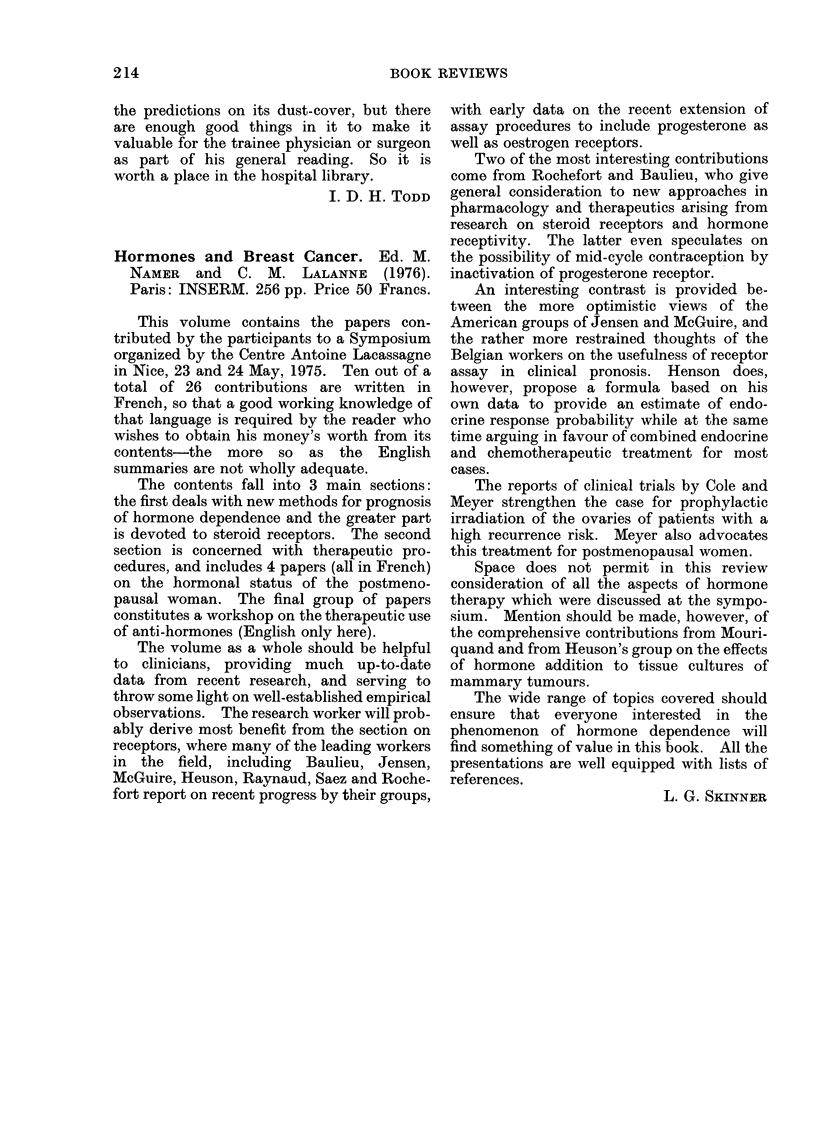# Hormones and Breast Cancer

**Published:** 1976-08

**Authors:** L. G. Skinner


**Hormones and Breast Cancer.** Ed. M. Namer and C. M. Lalanne (1976). Paris: INSERM. 256 pp. Price 50 Francs.

This volume contains the papers contributed by the participants to a Symposium organized by the Centre Antoine Lacassagne in Nice, 23 and 24 May, 1975. Ten out of a total of 26 contributions are written in French, so that a good working knowledge of that language is required by the reader who wishes to obtain his money's worth from its contents—the more so as the English summaries are not wholly adequate.

The contents fall into 3 main sections: the first deals with new methods for prognosis of hormone dependence and the greater part is devoted to steroid receptors. The second section is concerned with therapeutic procedures, and includes 4 papers (all in French) on the hormonal status of the postmenopausal woman. The final group of papers constitutes a workshop on the therapeutic use of anti-hormones (English only here).

The volume as a whole should be helpful to clinicians, providing much up-to-date data from recent research, and serving to throw some light on well-established empirical observations. The research worker will probably derive most benefit from the section on receptors, where many of the leading workers in the field, including Baulieu, Jensen, McGuire, Heuson, Raynaud, Saez and Rochefort report on recent progress by their groups, with early data on the recent extension of assay procedures to include progesterone as well as oestrogen receptors.

Two of the most interesting contributions come from Rochefort and Baulieu, who give general consideration to new approaches in pharmacology and therapeutics arising from research on steroid receptors and hormone receptivity. The latter even speculates on the possibility of mid-cycle contraception by inactivation of progesterone receptor.

An interesting contrast is provided between the more optimistic views of the American groups of Jensen and McGuire, and the rather more restrained thoughts of the Belgian workers on the usefulness of receptor assay in clinical pronosis. Henson does, however, propose a formula based on his own data to provide an estimate of endocrine response probability while at the same time arguing in favour of combined endocrine and chemotherapeutic treatment for most cases.

The reports of clinical trials by Cole and Meyer strengthen the case for prophylactic irradiation of the ovaries of patients with a high recurrence risk. Meyer also advocates this treatment for postmenopausal women.

Space does not permit in this review consideration of all the aspects of hormone therapy which were discussed at the symposium. Mention should be made, however, of the comprehensive contributions from Mouriquand and from Heuson's group on the effects of hormone addition to tissue cultures of mammary tumours.

The wide range of topics covered should ensure that everyone interested in the phenomenon of hormone dependence will find something of value in this book. All the presentations are well equipped with lists of references.

L. G. Skinner